# Association of Adverse Experiences and Exposure to Violence in Childhood and Adolescence With Inflammatory Burden in Young People

**DOI:** 10.1001/jamapediatrics.2019.3875

**Published:** 2019-11-04

**Authors:** Line Jee Hartmann Rasmussen, Terrie E. Moffitt, Louise Arseneault, Andrea Danese, Jesper Eugen-Olsen, Helen L. Fisher, HonaLee Harrington, Renate Houts, Timothy Matthews, Karen Sugden, Benjamin Williams, Avshalom Caspi

**Affiliations:** 1 Department of Psychology and Neuroscience, Duke University, Durham, North Carolina; 2 Clinical Research Centre, Copenhagen University Hospital Amager and Hvidovre, Hvidovre, Denmark; 3 Department of Psychiatry and Behavioral Sciences, Duke University School of Medicine, Durham, North Carolina; 4 Center for Genomic and Computational Biology, Duke University, Durham, North Carolina; 5 Social, Genetic, and Developmental Psychiatry Centre, Institute of Psychiatry, Psychology, and Neuroscience, King’s College London, London, United Kingdom; 6 Department of Child and Adolescent Psychiatry, Institute of Psychiatry, Psychology, and Neuroscience, King’s College London, London, United Kingdom; 7 National and Specialist Child and Adolescent Mental Health Services Trauma, Anxiety, and Depression Clinic, South London and Maudsley National Health Service Foundation Trust, London, United Kingdom

## Abstract

**Importance:**

Childhood stress exposure is associated with inflammation as measured by C-reactive protein (CRP) and interleukin 6 (IL-6). However, findings are inconsistent and effect sizes are small. The addition of soluble urokinase plasminogen activator receptor (suPAR), a new biomarker of chronic inflammation, may improve measurement of stress-related inflammatory burden.

**Objectives:**

To assess whether exposure to adverse experiences, stress, and violence is associated with an increase in suPAR levels in young people and to test the hypothesis that measuring suPAR in addition to CRP or IL-6 levels improves the assessment of the inflammatory burden associated with early-life stress.

**Design, Setting, and Participants:**

This cohort study included 1391 participants from a 1994 to 1995 birth cohort of twins from the nationally representative Environmental Risk Longitudinal Twin Study in the United Kingdom. Participants were followed up until 18 years of age (93% retention). Plasma samples were analyzed in July 2018, and statistical analysis was performed from October 1, 2018, to May 31, 2019.

**Exposures:**

Adverse childhood experiences and childhood and adolescent experience of stress and violence exposure.

**Main Outcomes and Measures:**

Plasma CRP, IL-6, and suPAR levels at 18 years of age.

**Results:**

Among 1391 young people (mean [SD] age, 18.4 [0.36] years; 733 [52.7%] female), those who had been exposed to stressful experiences had elevated suPAR levels by 18 years of age after controlling for sex, body mass index, and smoking: 0.03-ng/mL (95% CI, 0.01-0.05 ng/mL) increase in suPAR per each additional adverse childhood experience, 0.09-ng/mL (95% CI, 0.01-0.17 ng/mL) increase in suPAR per each additional severe childhood experience of stress or violence, and 0.04-ng/mL (95% CI, −0.02 to 0.10 ng/mL) increase in suPAR per each additional severe adolescent experience of stress or violence. Individuals exposed to multiple types of violence in both childhood and adolescence had 0.26-ng/mL (95% CI, 0.07-0.45 ng/mL) higher suPAR levels compared with children who did not experience stress or violence. These stress-exposed young people were significantly more likely to have elevated suPAR levels at 18 years of age even if they did not have elevated CRP or IL-6 levels. Measuring suPAR in addition to CRP or IL-6 increased the association between stress exposure and inflammatory burden. For example, after adjusting for CRP and IL-6 levels, each additional adverse childhood experience was associated with a 0.05-mL (95% CI, 0.03-0.07 ng/mL) increase in suPAR, each additional severe childhood experience of stress or violence was associated with a 0.14-ng/mL (95% CI, 0.06-0.22 ng/mL) increase in suPAR, and each additional severe adolescent experience of stress or violence was associated with a 0.10-ng/mL (95% CI, 0.04-0.16 ng/mL) increase in suPAR.

**Conclusions and Relevance:**

The results suggest that adult inflammation is associated with childhood exposure to stress. Adding information about suPAR to traditional biomarkers of inflammation may improve the measurement of inflammatory burden associated with exposure to stress and violence.

## Key Points

**Question:**

Is exposure to adverse experiences, stress, and violence in childhood associated with an increase in blood levels of the inflammatory biomarker soluble urokinase plasminogen activator receptor in young people?

**Findings:**

In this cohort study of 1391 young people followed up to 18 years of age in the United Kingdom, exposure to adverse experiences, stress, and violence during childhood or adolescence was associated with elevated levels of the soluble urokinase plasminogen activator receptor at 18 years of age, even in children who did not have elevated C-reactive protein or interleukin 6 levels.

**Meaning:**

The findings suggest that stress-related inflammation begins at a relatively young age, and the measurement of this inflammatory burden may be improved by adding information about soluble urokinase plasminogen activator receptor to traditional biomarkers of inflammation.

## Teaser

This cohort study assesses whether exposure to adverse experiences, stress, and violence is associated with an increase in soluble urokinase plasminogen activator receptor blood levels in young people.

## Introduction

Exposure to adverse experiences, stress, and violence during childhood and adolescence is associated with elevated risk of physical and mental health problems in adulthood,^1^ many of which have inflammatory origins. Inflammation could constitute one of the underlying mechanisms responsible for the biological embedding of childhood stress, indicating an association between exposure to early-life adversity and adverse health outcomes in later life.^2^

C-reactive protein (CRP) and interleukin 6 (IL-6) are among the most commonly measured biomarkers of inflammation.^3^ Different types of adverse experiences have been associated with increased CRP and IL-6 levels,^4^ including maltreatment,^5,6^ bullying,^7,8,9^ and sexual abuse.^10^ However, findings are not consistent; several studies report nonsignificant associations,^4,11,12^ and not all associations were found after controlling for the confounding effects of smoking or obesity.^10,13^ The Dunedin Longitudinal Study^14^ recently found that exposure to childhood risk factors, including adverse childhood experiences (ACEs), was associated with higher levels of a novel biomarker of inflammation, soluble urokinase plasminogen activator receptor (suPAR), in adults independently of smoking and body mass index (BMI).

suPAR is released to the bloodstream during proinflammatory conditions when the membrane-bound receptor uPAR is cleaved from the surface of immunologically active cells.^15^ Plasma levels of suPAR are thought to reflect a person’s overall level of immune activity. Elevated suPAR levels are observed in many diseases and pathologic conditions^16,17,18,19^ and are associated with development and progression of disease, adverse clinical outcomes, and mortality.^20,21^ High suPAR levels are also positively correlated with CRP and IL-6 in general and patient populations.^14,22^ Whereas CRP is an acute-phase reactant and a marker of acute inflammation and infections,^23^ suPAR appears to be less affected by acute conditions.^24^ Of interest, suPAR is associated with disease and mortality independent of CRP,^21,25^ the criterion standard marker of inflammation, suggesting that combined use of CRP and suPAR may provide a more accurate estimate of inflammatory burden by combining information about acute and chronic inflammation. Less is known about how suPAR compares with IL-6. Interleukin 6 is an important cytokine with proinflammatory or anti-inflammatory properties depending on the specific immunologic context. In addition to the immunologic functions of IL-6 in the acute-phase response, infections, inflammation, and cancer, IL-6 exerts multiple pleiotropic effects on other cell types, thereby regulating metabolism, hematopoiesis, and the neuroendocrine system.^26^ Although suPAR has been found to be associated with clinical outcomes independent of IL-6,^25^ whether the combined use of IL-6 and suPAR also provides additive information about inflammatory burden is unknown.

This report extends a previous study^14^ by investigating the association between suPAR and exposure to adverse experiences, stress, and violence during childhood and adolescence in the population-representative Environmental Risk (E-Risk) Longitudinal Twin Study followed up to 18 years of age. We assessed multiple types of adverse experiences during childhood and adolescence and cumulatively across the first 2 decades of life inside and outside the family, including emotional, physical, and sexual abuse; emotional and physical neglect; peer bullying; cyber bullying; and crime violence. In addition to evaluating these experiences in relation to suPAR, we compared the association of stress and violence exposure with CRP and IL-6 levels and tested whether adding suPAR to the measurement of CRP or IL-6 levels provides additional information about inflammation associated with stress and violence exposure.

## Methods

### Sample

This cohort study included members of the E-Risk Longitudinal Twin Study, which tracks the development of a 1994-1995 birth cohort of 2232 British children.^26^ In brief, the E-Risk Longitudinal Twin Study sample was constructed from 1999 to 2000, when 1116 of 1203 eligible families (92.8%) with same-sex 5-year-old twins participated in home-visit assessments. This sample comprises 1242 (55.7%) monozygotic (MZ) and 990 (44.4%) dizygotic (DZ) twins; sex was evenly distributed within zygosity (1140 [51.1%] female). Home visits were conducted when participants were aged 5, 7, 10, 12, and, most recently, 18 years of age (n = 2066 [92.6%]). At 18 years of age, each twin was interviewed by a different interviewer. The Joint South London and Maudsley and the Institute of Psychiatry research ethics committee approved each phase of the study. Parents gave written informed consent, and twins gave oral assent between 5 and 12 years of age and then written informed consent at 18 years of age. Plasma samples were analyzed in July 2018, and statistical analysis was performed from October 1, 2018, to May 31, 2019.

The sample represents socioeconomic conditions in the United Kingdom, as reflected in the families’ distribution on a neighborhood-level socioeconomic index (ACORN [A Classification of Residential Neighborhoods], developed by CACI Inc for commercial use): 25.6% of E-Risk Longitudinal Twin Study families live in wealthy achiever neighborhoods compared with 25.3% nationwide, 5.3% vs 11.6% live in urban prosperity neighborhoods, 29.6% vs 26.9% live in comfortably off neighborhoods, 13.4% vs 13.9% live in moderate means neighborhoods, and 26.1% vs 20.7% live in hard-pressed neighborhoods. The E-Risk Longitudinal Twin Study underrepresents urban prosperity neighborhoods because such households are often childless.

### Exposure to Adverse Experiences in Childhood and Adolescence

We assessed 4 forms of stressful experiences in childhood: (1) ACEs, as introduced by the US Centers for Disease Control and Prevention ACEs Study^1^ and expanded by the Philadelphia Urban ACE Survey^27^; (2) exposure to 6 types of severe childhood experiences of stress or violence between birth to 12 years of age; (3) exposure to 7 types of severe adolescent experiences of stress or violence at 12 to 18 years of age; and (4) exposure to cumulative stress and violence experiences throughout the life, as determined by applying latent class analysis (LCA) to stress and violence experiences data in childhood and adolescence. These measures have been reported previously^28,29,30,31^ and are described in the Box and detailed in eMethods 1 to 4 in the Supplement.

Box. **Description of Study Measures**

**ACEs**

Twenty ACEs were measured during childhood and adolescence up to 17 years of age: 10 conventional ACEs, corresponding to the 10 subcategories of childhood adversities introduced by the CDC Adverse Childhood Experiences Study,^1^ and 10 expanded ACEs, identified from the results from the Philadelphia Urban ACE Survey and routine activity theory, as previously described.^28^ The 10 conventional ACEs included physical abuse, sexual abuse, emotional abuse, physical neglect, emotional neglect, domestic violence exposure, household substance abuse, family history of mental illness, loss of a parent (parental death, separation, or divorce), and parental antisocial behavior. The 10 expanded ACEs included experiencing bullying, living in foster care, low childhood socioeconomic status, peer substance use, low parental monitoring (as evaluated by parents), low parental monitoring (as evaluated by children), participant-perceived unsafe neighborhood, high neighbor crime violence measured via neighbor survey, neighborhood rated as unsafe through systematic social observation, and high-crime neighborhood measured through official police records. Measurement details are provided in eMethods 1 in the Supplement. Among children in this study, 197 (14.2%) had no ACEs, 238 (17.1%) had 1 ACE, 229 (16.5%) had 2 ACES, and 727 (52.3%) had 3 or more ACES.

**Severe Childhood Experiences of Stress or Violence**

Exposure to 6 types of severe childhood experiences of stress or violence was assessed repeatedly when the children were 5, 7, 10, and 12 years of age, including exposure to domestic intimate partner violence between the mother and her partner, frequent bullying by peers, physical maltreatment by an adult, sexual abuse, emotional abuse and neglect, and physical neglect. Exposures were coded from 12-year dossiers for each child that comprised information from home visit staff, mothers, children, family physicians, and child protection interventions. Each exposure during childhood was coded on a 3-point scale (0 indicating no exposure, 1 indicating probable or less severe exposure, and 2 indicating definite or severe exposure). Following the guidelines by Finkelhor et al,^32^ we operationalized severe childhood experiences of stress or violence as the total number of adverse event types experienced by a child. All severe childhood experiences or stress or violence were summed. Measurement details are provided in eMethods 2 in the Supplement. Among children in this study, 1004 (72.2%) had no severe childhood experiences of stress or violence, 298 (21.4%) had 1 experience, 59 (4.2%) had 2 experiences, and 30 (2.2%) had 3 or more experiences.

**Severe Adolescent Experiences of Stress or Violence**

Severe adolescent experiences of stress or violence between the ages of 12 and 18 years were assessed at age 18 years of age when the twins were interviewed using the JVQ-R2,^33,34^ adapted as a clinical interview.^29^ The adapted JVQ-R2 comprised 45 questions covering 7 different forms of adverse experience: crime violence, peer or sibling violence, cyber bullying, sexual abuse, maltreatment, family violence, and neglect. Like severe childhood experiences of stress or violence experiences, each exposure during adolescence was coded on a 3-point scale (0 indicating no exposure, 1 indicating probable or less severe exposure, and 2 indicating definite or severe exposure). Severe adolescent experiences of stress or violence were derived by summing the number of severe adolescent experiences of stress or violence. Measurement details are provided in eMethods 3 in the Supplement. In this study, 887 adolescents (63.8%) had no severe adverse experiences, 275 (19.8%) had 1 experience, 131 (9.4%) had 2 experiences, and 98 (7.0%) had 3 or more experiences.

**Cumulative Stress and Violence Experiences**

Three groups of stress or violence experiences were identified with latent class analysis combining the 6 childhood and the 7 adolescent measures of severe experiences of stress or violence, as previously described.^31^ The latent class analysis classified participants into groups based on the degree of each participant’s exposure (none, moderate, or severe), and the analysis was performed using only participants who experienced at least 1 form of stress or violence experience. The 3 groups identified were (1) individuals who were exposed primarily to parental intimate partner violence during childhood (n = 213 [15.3%]), (2) individuals who were mainly bullied by peers and experienced street crime during childhood and adolescence (n = 354 [25.4%]), and (3) individuals who experienced multiple types of violence during childhood and adolescence (n = 129 [9.3%]). Measurement details are provided in eMethods 4 in the Supplement.

Abbreviations: ACEs, adverse childhood experiences; CDC, Centers for Disease Control and Prevention; JVQ-R2, Juvenile Victimization Questionnaire, second revision.

### Inflammatory Biomarkers

Venous blood was collected from 1700 of the 2066 participants (82.3%) with EDTA tubes. Tubes were spun at 2500*g* for 10 minutes and plasma samples obtained. Samples were stored at −80°C. Plasma samples were available for 1448 participants. Plasma CRP (high-sensitivity CRP) was measured using enzyme-linked immunosorbent assay (ELISA) (Quantikine ELISA Kit DCRP00, R&D Systems) following the manufacturer’s protocol. The coefficient of variation was 5.6%. Plasma IL-6 levels were measured using ELISA (Quantikine HS ELISA Kit HS600C, R&D Systems) following the manufacturer’s protocol. The coefficient of variation was 12.6%. Plasma suPAR levels were analyzed using ELISA (suPARnostic AUTO Flex ELISA, ViroGates A/S) following the manufacturer’s protocol. The coefficient of variation was 6%.

### Other Variables Associated With Inflammation

When participants were 18 years of age, we recorded BMI ( calculated as weight in kilograms divided by height in meters squared), body temperature, current daily smoking, current illness and injury (eTable 1 in the Supplement), and use of anti-inflammatory medication (corticosteroids) within the past 2 weeks. Children exposed to ACEs or stress and violence may also be exposed to unsanitary homes, which could be associated with elevated suPAR levels and potentially confound associations between ACEs or stress and violence exposure and suPAR level. The cleanliness of the homes was assessed when children were 12 years by home visitors answering the question, “Are visible rooms of the house clean?” (no, somewhat, or yes).

Childhood socioeconomic status (SES) was defined through a standardized composite of parental income, educational level, and occupation. The 3 SES indicators were highly correlated (*r* = 0.57-0.67) and loaded onto 1 latent factor. The population-wide distribution of the resulting factor was divided in tertiles for analyses.^35^

### Statistical Analysis

Both CRP and IL-6 levels were log-transformed to improve normality of their distributions, as commonly done.^36^ Distributions of CRP, IL-6, and suPAR levels are shown in the eFigure in the Supplement. Sex-adjusted regression coefficients were calculated to test associations between the inflammatory biomarkers with clinical characteristics of the sample. The association between ACEs or stress and violence exposure and inflammation was tested using ordinary least squares regression, with continuous measures of CRP, IL-6, and suPAR levels. Models were adjusted for sex, BMI, and smoking. Analyses of suPAR levels were adjusted for cleanliness of the home, childhood SES, CRP level, or IL-6 level. We report unstandardized B and standardized β coefficients, with 95% CIs adjusted to control for the nonindependence of observations of twins within families. Estimates and 95% CIs for log-transformed variables were back-transformed by exponentiating.

To analyze associations of ACEs or stress and violence exposure with combined CRP (untransformed values) and suPAR levels or combined IL-6 (untransformed values) and suPAR levels, we created groups characterized by high or low levels of CRP and suPAR or IL-6 and suPAR, as previously described.^14^ For CRP, we used the established clinical cutoff (3 mg/L [to convert to nanomoles per liter, multiply by 9.524]) to identify participants with high CRP levels; thus, high CRP level indicates a CRP level greater than 3 mg/L (n = 287 [20.6%]). Clinical cutoffs for suPAR and IL-6 have not yet been established. To identify participants with high suPAR or high IL-6 levels, we chose a cutoff for each that corresponded to a similar percentage as high CRP level; thus, high suPAR level indicates a suPAR level greater than 3.81 ng/mL (n = 286 [20.6%]), and high IL-6 level indicates an IL-6 level greater than 1.48 pg/mL (n = 286 [20.6%]). We created 4 groups of individuals characterized by (1) low CRP and low suPAR levels (n = 920 [66.1%]), (2) high CRP and low suPAR levels (n = 185 [13.3%]), (3) low CRP and high suPAR levels (n = 184 [13.2%]), and (4) high CRP and high suPAR levels (n = 102 [7.3%]). Similarly, we created 4 groups of individuals characterized by (1) low IL-6 and low suPAR levels (n = 916 [65.9%]), (2) high IL-6 and low suPAR levels (n = 189 [13.6%]), (3) low IL-6 and high suPAR levels (n = 189 [13.6%]), and (4) high IL-6 and high suPAR levels (n = 97 [7.0%]).

In addition, we performed an LCA that combined ln(CRP), ln(IL-6), and suPAR to classify participants into groups based on each participant’s levels of the 3 biomarkers, accounting for clustering of twins within families. The LCA identified 3 inflammation groups of individuals (eTable 2 and eTable 3 in the Supplement): low levels of all 3 biomarkers, elevated CRP and IL-6 levels, and elevated CRP, IL-6, and suPAR levels. The association between stress and violence exposure and the inflammation groups was tested using multinomial logistic regression, reporting odds ratios (ORs) with 95% CIs. Two-sided *P* < .05 was a priori designated statistically significant.

## Results

Of the 2066 children participating at 18 years of age, 1419 (68.7%) had complete data for childhood and adolescent adverse experiences; CRP, IL-6, and suPAR measurements at 18 years of age; and the covariates BMI and smoking. Participants with levels greater than 4 SDs above the means of CRP (n = 18), IL-6 (n = 7), or suPAR (n = 3) levels were excluded, leaving a final sample of 1391 (mean [SD] age, 18.4 [0.36] years; 733 [52.7%] female). No significant differences were found for those with complete data included in this article vs those without in terms of mean SES (mean, 1.99 [95% CI, 1.95-2.04] vs 2.02 [95% CI, 1.97-2.08]; *P* = .47), ACEs (mean, 3.22 [95% CI, 3.08-3.36] vs 3.04 [95% CI, 2.86-3.21]; *P* = .17), severe childhood experiences of stress or violence (mean, 0.36 [95% CI, 0.33-0.40] vs 0.34 [95% CI, 0.29-0.39]; *P* = .49), or severe adolescent experiences of stress or violence (mean, 0.60 [95% CI, 0.55-0.65] vs 0.55 [95% CI, 0.49-0.62]; *P* = .34).

Correlates of CRP, IL-6, and suPAR are given in Table 1. Compared with male participants, female participants had higher levels of CRP (*r* = 0.14; 95% CI, 0.08-0.20; *P* < .001) and suPAR (*r* = 0.23; 95% CI, 0.17-0.29; *P* < .001) but not IL-6 (r = 0.002; 95% CI, −0.06 to 0.06; *P* = .95). Participants with high BMIs had higher levels of all 3 inflammatory biomarkers (CRP: *r* = 0.35; 95% CI, 0.30-0.40; *P* < .001; IL-6: *r* = 0.19; 95% CI, 0.13-0.24; *P* < .001; and suPAR: *r* = 0.29; 95% CI, 0.22-0.35; *P* < .001). Tobacco smoking was associated with elevated levels of IL-6 (*r* = 0.06; 95% CI, 0.005-0.12; *P* = .03) and suPAR (*r* = 0.22; 95% CI, 0.16-0.28; *P* < .001) but not CRP (*r* = 0.04; 95% CI, −0.02 to 0.09; *P* = .20). In contrast, acute conditions, such as body temperature and current illness or injury (Table 1 and eTable 1 in the Supplement), were associated with elevated levels of CRP (body temperature: *r* = 0.06; 95% CI, 0.004-0.12; *P* = .04; current illness or injury: *r* = 0.18; 95% CI, 0.12-0.23; *P* < .001) and IL-6 (current illness or injury: *r* = 0.14; 95% CI, 0.08-0.20; *P* < .001) but not suPAR (body temperature: *r* = 0.03; 95% CI, −0.03 to 0.09; *P* = .31; current illness or injury: *r* = 0.02; 95% CI, −0.03 to 0.08; *P* = .36). Use of anti-inflammatory medication (corticosteroids) in the past 2 weeks was not associated with any of the inflammatory biomarkers (CRP: *r* = 0.01; 95% CI, −0.04 to 0.06; *P* = .65: IL-6: *r* = −0.04; 95% CI, −0.15 to 0.07; *P* = .46; suPAR: *r* = 0.008; 95% CI, −0.07 to 0.08; *P* = .83). The 3 inflammatory biomarkers (CRP, IL-6, and suPAR) were positively intercorrelated (*r* = 0.25–0.39) (Table 1).

**Table 1.** Sex-Adjusted Correlates of Plasma CRP, IL-6, and suPAR at 18 Years of Age in the E-Risk Longitudinal Twin Study

Variable	Total	CRP^a^		IL-6^a^		suPAR	
		*r* (95% CI)^b^	*P* Value	*r* (95% CI)^b^	*P* Value	*r* (95% CI)^b^	*P* Value
Correlates of inflammation							
Female, No./total No. (%)	733/1391 (52.7)	0.14 (0.08 to 0.20)	<.001	0.002 (−0.06 to 0.06)	.95	0.23 (0.17 to 0.29)	<.001
BMI, mean (SE)	22.9 (0.15)	0.35 (0.30 to 0.40)	<.001	0.19 (0.13 to 0.24)	<.001	0.29 (0.22 to 0.35)	<.001
Daily smoking, No./total No. (%)	315/1391 (22.6)	0.04 (−0.02 to 0.09)	.20	0.06 (0.005 to 0.12)	.03	0.22 (0.16 to 0.28)	<.001
Body temperature, mean (SE), °C	36.3 (0.02)	0.06 (0.004 to 0.12)	.04	0.06 (−0.01 to 0.12)	.07	0.03 (−0.03 to 0.09)	.31
Current illness or injury, No./total No. (%)^c^	323/1390 (23.2)	0.18 (0.12 to 0.23)	<.001	0.14 (0.08 to 0.20)	<.001	0.02 (−0.03 to 0.08)	.36
Anti-inflammatory medication use, No./total No. (%)^d^	17/1391 (1.2)	0.01 (−0.04 to 0.06)	.65	−0.04 (−0.15 to 0.07)	.46	0.008 (−0.07 to 0.08)	.83
Cleanliness of home, No./total No. (%)	107/1342 (8.0)^e^	−0.04 (−0.10 to 0.02)	.22	−0.07 (−0.12 to −0.005)	.03	−0.11 (−0.17 to −0.05)	<.001
Socioeconomic status, No./total No. (%)	466/1391 (33.5)^f^	−0.05 (−0.11 to 0.01)	.09	−0.10 (−0.16 to −0.03)	.002	−0.16 (−0.22 to −0.10)	<.001
Inflammatory biomarkers, mean (SE)							
CRP level, mg/L^a^	2.34 (0.11)	NA	NA	0.39 (0.33 to 0.44)	<.001	0.25 (0.20 to 0.31)	<.001
IL-6 level, pg/mL^a^	1.19 (0.03)	0.39 (0.33 to 0.44)	<.001	NA	NA	0.27 (0.21 to 0.33)	<.001
suPAR level, ng/mL	3.23 (0.03)	0.25 (0.20 to 0.31)	<.001	0.27 (0.21 to 0.33)	<.001	NA	NA

Abbreviations: BMI, body mass index (calculated as weight in kilograms divided by height in meters squared); CRP, C-reactive protein; E-Risk, Environmental Risk; IL-6, interleukin 6; NA, not applicable; suPAR, soluble urokinase plasminogen activator receptor.

SI conversion factor: To convert CRP to nanomoles per liter, multiply by 9.524.

^a^ Log-transformed (natural logarithm).

^b^ Standardized estimated regression coefficients; all correlations were adjusted for sex, and *P* values were adjusted for clustering within families.

^c^ The current illness or injury index is a count of 16 conditions present on the day of blood sample obtainment. Correlations with individual illnesses and injuries are provided in eTable 1 in the Supplement.

^d^ Any use of anti-inflammatory medication (corticosteroids) in the past 2 weeks.

^e^ Number of homes reported to be unclean by home visitors when children were 12 years of age. A total of 205 (15.3%) were reported as somewhat clean, and 1030 (76.8%) were reported as clean.

^f^ Number of families scored as low on the social class composite. A total of 469 (33.7%) were scored as middle social class, and 456 (32.8%) were scored as high social class.

### Association of Adverse Experiences With Inflammatory Biomarker Levels

Children exposed to more ACEs had higher levels of suPAR and IL-6 but not CRP at 18 years of age. Only the association with suPAR remained after adjustment for sex, BMI, and smoking (CRP: B = 1.00; 95% CI, 0.97-1.03; IL-6: B = 1.01; 95% CI, 1.00-1.03; suPAR: B = 0.03; 95% CI, 0.01-0.05) (Table 2). In general, adverse experiences were most strongly associated with suPAR at 18 years of age. Participants who experienced stress and violence exposure as children had higher levels of suPAR and IL-6 but not CRP even after controlling for sex, BMI, and smoking (suPAR: B = 0.09, 95% CI, 0.01-0.17; IL-6: B = 1.06; 95% CI, 1.01-1.12; CRP: B = 1.04; 95% CI, 0.92-1.17) (Table 2). Participants who experienced stress and violence exposure as adolescents had higher levels of suPAR and CRP but not IL-6 (suPAR: B = 0.11; 95% CI, 0.05-0.18; CRP: B = 1.09, 95% CI, 1.00-1.18; IL-6: B = 1.02; 95% CI, 0.99-1.06), but none of these associations remained after adjustment for covariates (Table 2). Participants exposed to cumulative adverse experiences across childhood and adolescence had elevated suPAR levels. In particular, levels of suPAR (but not CRP or IL-6) were elevated among those exposed to domestic violence (suPAR: B = 0.25, 95% CI, 0.10-0.40; CRP: B = 1.04; 95% CI, 0.83-1.29; IL-6: B = 1.01; 95% CI, 0.91-1.13) and those who experienced multiple types of violence in childhood and adolescence (suPAR: B = 0.26; 95% CI, 0.07-0.45; CRP: B = 1.06; 95% CI, 0.81-1.38; IL-6: B = 1.02; 95% CI, 0.88-1.18) even after controlling for sex, BMI, and smoking (Table 2).

**Table 2.** Associations of Childhood Adversities With Plasma CRP, IL-6, and suPAR Levels at 18 Years of Age in 1391 Participants in the E-Risk Longitudinal Twin Study

Measure	CRP^a^				IL-6^a^				suPAR			
	Unadjusted		Adjusted^b^		Unadjusted		Adjusted^b^		Unadjusted		Adjusted^b^	
	B (95% CI)^c^^,^^d^	β (95% CI)^e^	B (95% CI)^c^^,^^d^	β (95% CI)^e^	B (95% CI)^c^^,^^d^	β (95% CI)^e^	B (95% CI)^c^^,^^d^	β (95% CI)^e^	B (95% CI)^c^	β (95% CI)^e^	B (95% CI)^c^	β (95% CI)^e^
Adverse childhood experiences	1.01 (0.98 to 1.04)	0.02 (−0.04 to 0.08)	1.00 (0.97 to 1.03)	0.002 (−0.06 to 0.06)	1.02 (1.01 to 1.03)	0.08 (0.02 to 0.13)	1.01 (1.00 to 1.03)	0.06 (−0.01 to 0.12)	0.05 (0.02 to 0.07)	0.13 (0.06 to 0.19)	0.03 (0.01 to 0.05)	0.08 (0.02 to 0.15)
Childhood experiences of stress or violence	1.07 (0.95 to 1.22)	0.03 (−0.03 to 0.09)	1.04 (0.92 to 1.17)	0.02 (−0.04 to 0.08)	1.08 (1.03 to 1.14)	0.09 (0.03 to 0.14)	1.06 (1.01 to 1.12)	0.07 (0.01 to 0.13)	0.15 (0.06 to 0.24)	0.11 (0.05 to 0.17)	0.09 (0.01 to 0.17)	0.07 (0.01 to 0.12)
Adolescent experiences of stress or violence	1.09 (1.00 to 1.18)	0.05 (−0.002 to 0.11)	1.05 (0.97 to 1.14)	0.03 (−0.02 to 0.09)	1.02 (0.99 to 1.06)	0.03 (−0.02 to 0.09)	1.01 (0.97 to 1.05)	0.01 (−0.05 to 0.07)	0.11 (0.05 to 0.18)	0.11 (0.05 to 0.18)	0.04 (−0.02 to 0.10)	0.04 (−0.02 to 0.10)
**Groups of Cumulative Experiences of Stress or Violence**												
Exposure to parental intimate partner violence in childhood^f^	1.10 (0.87 to 1.40)	0.07 (−0.10 to 0.24)	1.04 (0.83 to 1.29)	0.03 (−0.13 to 0.18)	1.04 (0.93 to 1.16)	0.07 (−0.11 to 0.24)	1.01 (0.91 to 1.13)	0.02 (−0.15 to 0.20)	0.33 (0.17 to 0.49)	0.36 (0.19 to 0.53)	0.25 (0.10 to 0.40)	0.27 (0.11 to 0.43)
Exposure to peer and street crime stress and violence during childhood and adolescence^f^	1.20 (1.00 to 1.44)	0.13 (−0.003 to 0.26)	1.06 (0.89 to 1.27)	0.04 (−0.08 to 0.17)	1.02 (0.94 to 1.11)	0.04 (−0.09 to 0.17)	0.99 (0.91 to 1.07)	−0.02 (−0.15 to 0.11)	0.16 (0.03 to 0.29)	0.17 (0.03 to 0.31)	0.02 (−0.10 to 0.13)	0.02 (−0.11 to 0.14)
Exposure to multiple types of violence during childhood and adolescence^f^	1.14 (0.86 to 1.52)	0.10 (−0.11 to 0.30)	1.06 (0.81 to 1.38)	0.04 (−0.16 to 0.23)	1.07 (0.93 to 1.23)	0.11 (−0.12 to 0.33)	1.02 (0.88 to 1.18)	0.03 (−0.20 to 0.26)	0.43 (0.22 to 0.64)	0.47 (0.24 to 0.69)	0.26 (0.07 to 0.45)	0.28 (0.08 to 0.48)

Abbreviations: CRP, C-reactive protein; E-Risk, Environmental Risk; IL-6, interleukin 6; suPAR, soluble urokinase plasminogen activator receptor.

^a^ Log-transformed (natural logarithm).

^b^ Adjusted for sex, body mass index, and smoking.

^c^ Unstandardized B coefficient for ordinary least squares regression model, in which a 1-unit change in the variable (eg, adverse childhood experiences) is associated with a corresponding change in B, holding all other variables constant.

^d^ Log-transformed estimates and 95% CIs were back-transformed by exponentiating.

^e^ Standardized β coefficients.

^f^ Estimates represent mean differences from the no adverse experience group.

Correlations among different types of adverse experiences (eg, physical abuse, street crime, and cyber bullying) and CRP, IL-6, and suPAR levels are presented in eTable 4 in the Supplement. suPAR was associated with various forms of adverse experiences, suggesting that the elevation in suPAR levels among young people who underwent adverse experiences was not specific to any particular type of adverse experience but was rather a function of cumulative exposure.

Home visitor ratings allowed us to investigate whether living in a nonhygienic home during childhood and adolescence explained the association between adverse experiences and inflammation. Cleanliness of the home was negatively correlated with levels of suPAR (*r* = −0.11; 95% CI, −0.17 to −0.05; *P* < .001) and IL-6 (*r* = −0.07; 95% CI, −0.12 to −0.005; *P* = .03) but not CRP (*r* = −0.04; 95% CI, −0.10 to 0.02; *P* = .22) (Table 1). When controlling for cleanliness of the home, all adverse experience exposures remained associated with elevated suPAR level at 18 years of age (eTable 5 in the Supplement).

Childhood SES was negatively correlated with levels of suPAR (*r* = −0.16; 95% CI, −0.22 to −0.10; *P* < .001) and IL-6 (*r* = −0.10; 95% CI, −0.16 to −0.03; *P* = .002) but not CRP (*r* = −0.05; 95% CI, −0.11 to 0.01; *P* = .09) (Table 1). When controlling for SES, all adverse experience exposures remained associated with elevated suPAR level at 18 years of age (eTable 5 in the Supplement).

### Importance of suPAR in Adverse Experience–Associated Inflammation 

Adverse experiences were associated with suPAR apart from any association with CRP and IL-6 (eTable 5 in the Supplement). Measuring suPAR in addition to CRP or IL-6 increased the association between stress exposure and inflammatory burden. For example, after adjusting for CRP and IL-6 levels, each additional adverse childhood experience was associated with a 0.05-mL (95% CI, 0.03-0.07 ng/mL) increase in suPAR, each additional severe childhood experience of stress or violence was associated with a 0.14-ng/mL (95% CI, 0.06-0.22 ng/mL) increase in suPAR, and each additional severe adolescent experience of stress or violence was associated with a 0.10-ng/mL (95% CI, 0.04-0.16 ng/mL) increase in suPAR. Young people who were exposed to more ACEs, childhood stress and violence exposure, adolescent stress and violence exposure, and cumulative stress and violence exposure were significantly more likely to have elevated levels of both CRP and suPAR at 18 years of age (ACEs: OR, 1.41; 95% CI, 1.16-1.71; *P* < .001; childhood stress and violence exposure: OR, 1.33; 95% CI, 1.12-1.57; *P* = .001; adolescent stress and violence exposure: OR, 1.28; 95% CI, 1.05-1.56; *P* = .02; cumulative stress and violence exposure: OR, 2.51; 95% CI, 1.27-4.97; *P* = .008) (Figure, A, and eTable 6 in the Supplement) as well as elevated levels of IL-6 and suPAR at 18 years of age (ACEs: OR, 1.39; 95% CI, 1.13-1.71; *P* = .002; childhood stress and violence exposure: OR, 1.34; 95% CI, 1.11-1.63; *P* = .003; adolescent stress and violence exposure: OR, 1.35; 95% CI, 1.12-1.64; *P* = .002; cumulative stress and violence exposure: OR, 3.04; 95% CI, 1.61-5.73; *P* < .001) (Figure, B, and eTable 6 in the Supplement).

**Figure.**
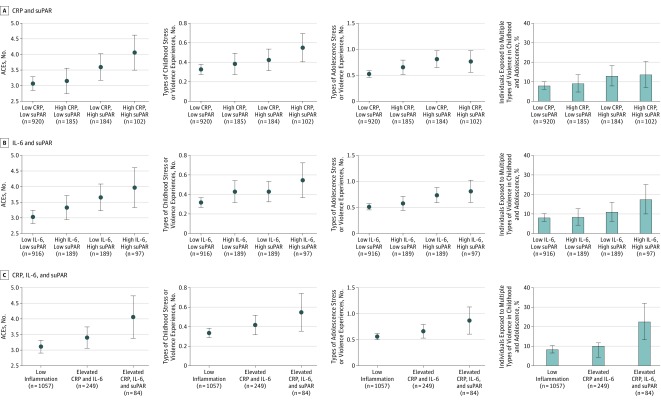
Frequency of Adverse Childhood Experiences (ACEs) and Severe Experiences of Stress or Violence in Childhood or Adolescence Stratified by Inflammatory Biomarker Levels Mean number of ACEs, number of types of adverse experiences during childhood, number of types of adverse experiences during adolescence, or percentage of young people exposed to multiple types of violence during childhood or adolescence for individuals of the Environmental Risk (E-Risk) Longitudinal Twin Study stratified in groups of high C-reactive protein (CRP) level (>3 mg/L) and/or high soluble urokinase plasminogen activator receptor (suPAR) level (>3.81 ng/mL) (A), high interleukin 6 (IL-6) level (>1.48 pg/mL) and/or high suPAR level (>3.81 ng/mL) (B); or inflammation groups identified by latent class analysis (ie, low inflammation, elevated CRP and IL-6 levels, and elevated CRP, IL-6, and suPAR levels) (C). Error bars indicate 95% CIs.

However, adverse experiences were also prominent in the group of participants with low CRP and high suPAR (or low IL-6 and high suPAR) levels, who would inadvertently have been misclassified as having low inflammation if suPAR levels had not been measured. Similarly, the LCA revealed that young people who had elevated suPAR levels at 18 years of age in addition to elevated CRP and IL-6 levels had been exposed to more ACEs and childhood, adolescent, and cumulative stress and violence experiences compared with those with low levels of all 3 inflammatory biomarkers or with those with mainly high levels of CRP and IL-6 but not suPAR (Figure, C, and eTable 7 in the Supplement).

## Discussion

In this 2-decade prospective cohort study, we tested the usefulness of a new biomarker, suPAR, to understand the biological association with stress in the first part of the life course. First, children exposed to adversities and to multiple forms of stress and violence during childhood and adolescence had elevated suPAR levels by the time they reached 18 years of age. The association could not be attributed to BMI, smoking, or living in unhygienic homes during that period. Second, suPAR levels appear to add information about the health implications of stressful experiences in childhood beyond the more established biomarkers CRP and IL-6. We observed the strongest associations between stress exposure and inflammation when combining biomarkers, and we also found that adverse experiences were prominent in the group of participants with low CRP or low IL-6 level, who would have inadvertently been assigned to the low inflammation group if suPAR levels had not been assayed. These results replicate findings from the Dunedin Longitudinal Study,^14^ which found that exposure to ACEs was associated with elevated levels of suPAR at midlife. Thus, suPAR may be a valuable adjunct to estimating inflammatory burden that may be associated with childhood stress exposure. This conclusion was supported by an LCA that revealed that young people with elevated inflammation could be separated into 2 categories: those with elevated CRP and IL-6 levels and those with elevated CRP, IL-6, and suPAR levels, the latter of which had stronger associations with adverse experiences.

Inflammation has been the target of investigation for researchers seeking to understand the biological variables associated with stress. Much of this work has relied on measuring CRP and IL-6 levels. A meta-analysis^4^ revealed significant associations between ACEs and levels of these 2 inflammatory biomarkers. However, effects were small, and more than half of the studies, many of which were well powered, did not reveal positive associations.

Part of the difficulty may be that traditional markers of inflammation mix historical and acute effects; for example, CRP and IL-6 are involved in the acute-phase response. suPAR, the soluble form of uPAR, has been put forward as a marker of chronic inflammation. The expression and shedding of uPAR are upregulated under inflammatory conditions and in response to immunologic stimuli.^37,38^ Thus, the suPAR level is elevated in many diseases with an inflammatory component,^21^ whereas it is generally low, although still detectable, in healthy individuals.^39^ Common risk factors for chronic disease, such as smoking and morbid obesity, are linked to elevated suPAR levels,^40^ and elevated suPAR levels are associated with development and progression of disease and adverse outcomes, including mortality.^20^ In contrast to many markers of inflammation, which are labile and rapidly upregulated and downregulated,^41,42^ suPAR appears to be more stable and less sensitive to acute influences^43^ and does not fluctuate with circadian rhythm.^44^ Of the 2 categories of elevated inflammation identified by the LCA in this study, the one with elevated suPAR in addition to CRP and IL-6 was more strongly associated with stress and violence exposure during childhood and adolescence than the one with mainly elevated CRP and IL-6 levels. This finding supports the conclusion that adding suPAR to CRP and IL-6 measurement may improve the assessment of chronic inflammation associated with early-life stress.

### Limitations

This study has limitations. First, plasma samples were only available for 1448 participants in the longitudinal study. However, no significant stress-exposure differences were found between participants who did and did not provide blood samples. Second, we studied twins, who may not represent singletons. However, the same pattern of associations between adverse experiences and suPAR levels was found in the Dunedin Longitudinal Study cohort of singletons.^14^ Third, although the distributional properties of suPAR are appealing for research purposes, the optimal threshold for its use as a diagnostic biomarker has not yet been determined. Fourth, the detected effect sizes were modest for suPAR, although this is to be expected in a sample of generally healthy young adults. Moreover, after we examined cumulative adverse experiences, effect sizes increased, a finding that underscores the importance of evaluating multiple risk factor exposures rather than any single exposure.^45^ Fifth, we collected inflammation data for the first time only in participants at 18 years of age, preventing us from analyzing the association of stress and violence exposure with inflammation trajectories over time. Longitudinal studies of suPAR are needed. In addition, randomized clinical trials of interventions intended to reduce effects of violence exposure should include inflammation biomarkers as outcome measures.^46^ One study^47^ found that favorable lifestyle changes were associated with reduced suPAR levels, suggesting suPAR as an outcome measure for studies of potential reversibility of chronic inflammation associated with early-life risk exposures. Sixth, we were able to identify risk factors associated with elevated suPAR levels, but because of the observational study design, we cannot rule out noncausal, alternative explanations of the associations.

## Conclusions

Inflammation has been suggested to fill the black box that connects childhood stress exposure to poor adult health, and it is under vigorous investigation.^48^ The results of the present study suggest that adult inflammation is associated with childhood stress exposure,^2^ with inflammation beginning by the time young people exposed to stress reach 18 years of age. Along with a previous report,^14^ the present study suggests that adding information about suPAR to traditional biomarkers of inflammation may improve the measurement of stress-related inflammatory burden.

## Notes

Supplement. eMethods 1. Assessment of Adverse Childhood Experiences (ACEs) eMethods 2. Assessment of Severe Childhood Experiences of Stress or Violence eMethods 3. Assessment of Severe Adolescent Experiences of Stress or Violence eMethods 4. Assessment of Cumulative Stress and Violence Experiences eFigure. Distributions of CRP, IL-6, and suPAR in the E-Risk Longitudinal Twin Study eTable 1. Correlations (Sex-Adjusted) of Plasma CRP, Plasma IL-6, and Plasma suPAR With Individual Illnesses or Injuries on the Day of Blood Sampling at Age 18 Years in the E-Risk Study (n = 1390) eTable 2. Results of a Latent Class Analysis Using Data About Inflammation Measured With CRP, IL-6, and suPAR (n = 1390) eTable 3. Levels of CRP, IL-6, and suPAR in the 3 Inflammation Groups Identified by Latent Class Analysis (n = 1390) eTable 4. Correlations Between Different Types of Adverse Experiences and CRP, IL-6, and suPAR at Age 18 Years in the E-Risk Study eTable 5. Associations of Childhood Adversities With Plasma suPAR at Age 18 Years in the E-Risk Study After Adjustment for Sex and Indicated Correlates eTable 6. Associations Between Adverse Experiences and Inflammation Groups at Age 18 Years eTable 7. Associations Between Adverse Experiences and Latent Class Inflammation Groups at Age 18 Years eReferences
