# Supplementary material for: Association of Adverse Experiences and Exposure to Violence in Childhood and Adolescence With Inflammatory Burden in Young People
Source: JAMA Pediatr. 2019 Nov 4;174(1):38–47. doi: 10.1001/jamapediatrics.2019.3875 (PMC6830440; doi:10.1001/jamapediatrics.2019.3875)
Supplement: Supplement. — eMethods 1. Assessment of Adverse Childhood Experiences (ACEs) eMethods 2. Assessment of Severe Childhood Experiences of Stress or Violence eMethods 3. Assessment of Severe Adolescent Experiences of Stress or Violence eMethods 4. Assessment of Cumulative Stress and Violence Experiences eFigure. Distributions of CRP, IL-6, and suPAR in the E-Risk Longitudinal Twin Study eTable 1. Correlations (Sex-Adjusted) of Plasma CRP, Plasma IL-6, and Plasma suPAR With Individual Illnesses or Injuries on the Day of Blood Sampling at Age 18 Years in the E-Risk Study (n = 1390) eTable 2. Results of a Latent Class Analysis Using Data About Inflammation Measured With CRP, IL-6, and suPAR (n = 1390) eTable 3. Levels of CRP, IL-6, and suPAR in the 3 Inflammation Groups Identified by Latent Class Analysis (n = 1390) eTable 4. Correlations Between Different Types of Adverse Experiences and CRP, IL-6, and suPAR at Age 18 Years in the E-Risk Study eTable 5. Associations of Childhood Adversities With Plasma suPAR at Age 18 Years in the E-Risk Study After Adjustment for Sex and Indicated Correlates eTable 6. Associations Between Adverse Experiences and Inflammation Groups at Age 18 Years eTable 7. Associations Between Adverse Experiences and Latent Class Inflammation Groups at Age 18 Years eReferences [file jamapediatr-174-38-s001.pdf]

Supplementary Online Content

Rasmussen, LJH, Moffitt TE, Arseneault L, et al. Association of adverse experiences and exposure to violence in childhood and adolescence with inflammatory burden in young people. *JAMA Pediatr*. Published online November 4, 2019. doi:10.1001/jamapediatrics.2019.3875

eMethods 1. Assessment of Adverse Childhood Experiences (ACEs)

eMethods 2. Assessment of Severe Childhood Experiences of Stress or Violence

eMethods 3. Assessment of Severe Adolescent Experiences of Stress or Violence

eMethods 4. Assessment of Cumulative Stress and Violence Experiences

eFigure. Distributions of CRP, IL-6, and suPAR in the E-Risk Longitudinal Twin Study

eTable 1. Correlations (Sex-Adjusted) of Plasma CRP, Plasma IL-6, and Plasma suPAR With Individual Illnesses or Injuries on the Day of Blood Sampling at Age 18 Years in the E-Risk Study (n = 1390)

eTable 2. Results of a Latent Class Analysis Using Data About Inflammation Measured With CRP, IL-6, and suPAR (n = 1390)

eTable 3. Levels of CRP, IL-6, and suPAR in the 3 Inflammation Groups Identified by Latent Class Analysis (n = 1390)

eTable 4. Correlations Between Different Types of Adverse Experiences and CRP, IL-6, and suPAR at Age 18 Years in the E-Risk Study

eTable 5. Associations of Childhood Adversities With Plasma suPAR at Age 18 Years in the E-Risk Study After Adjustment for Sex and Indicated Correlates

eTable 6. Associations Between Adverse Experiences and Inflammation Groups at Age 18 Years

eTable 7. Associations Between Adverse Experiences and Latent Class Inflammation Groups at Age 18 Years

eReferences

This supplementary material has been provided by the authors to give readers additional information about their work.

© 2019 Rasmussen LJH et al. JAMA Pediatrics.

© 2019 Rasmussen LJH et al. JAMA Pediatrics.

eMethods 1. Assessment of Adverse Childhood Experiences (ACEs)

We have previously reported on the measurement of ACEs.[1] Here we summarize the method.

We measured ACEs in two categories. First, we measured conventional ACEs:[2] physical abuse, sexual abuse, emotional abuse and neglect, physical neglect, domestic violence exposure, parental antisocial behavior, family history of substance abuse, family history of mental health disorders, and parental separation or divorce. Second, from the results of the Philadelphia ACEs survey, we identified 10 adversities that we could test as part of the E-Risk Study: experiencing bullying, living in foster care, low childhood socioeconomic status, peer substance abuse, low parental monitoring (as evaluated by parents), low parental monitoring (as evaluated by children), participant-perceived unsafe neighborhood, high neighborhood crime victimization (as evaluated by a survey of neighbors), neighborhood rated as unsafe (as evaluated by systematic social observation), and high-crime neighborhood (as evaluated by police records).

Physical abuse, sexual abuse, emotional abuse and neglect, physical neglect, and domestic violence were assessed as described below in the section on childhood victimization (eMethods 2 "Assessment of Severe Childhood Experiences of Stress or Violence"). Only severe cases of these measures (coded as 2; see below) were used for the assessment of ACEs.

Parental antisocial behavior. Father's and mother's history of antisocial behavior was reported by the mothers when the children were 5 years old. Mothers were interviewed using the Young Adult Behavior Checklist,[3] which was modified to obtain lifetime data. Full details of father's and mother's history of antisocial behavior within the E-Risk Study are reported elsewhere.[4] A study of mother–father agreement about men's antisocial behavior in this sample showed that women provided reliable information about the behavior of their children's father.[5] The variable was coded as a dichotomous indicator of approximately the top quarter of the distribution of antisocial behaviors of parents (n=560).

Family history of substance abuse. Substance use for any of the child's biological mother, father, maternal grandparents, or maternal aunts and uncles was reported by the mother when the children were 12 years old. Family history algorithms follow those outlined by Milne et al., 2008.[6] The variable was coded as a dichotomous indicator of approximately the top quarter of the proportion of family members with a history of substance use (n=478).

Family history of mental health disorders. Family history of a report of hospitalization for psychiatric disorder or attempted or completed suicide for any of the child's biological mother, father, maternal grandparents, or maternal aunts and uncles (for additional details see Belsky et al., 2012[7]). Family history of mental illness was reported for 658 of the children.

Parental separation or divorce. Biological parent separation or divorce was assessed at each interview, up to 10 years of age, by questions on whether the biological parents were living in the same household. Parental separation or divorce was reported for 1,024 of the children.

Bullying victimization. Severe bullying up to age 12 years was assessed from mother's or participant's reports and was operationally defined as evidence of (a) repeated harmful actions, (b) between children, and (c) where there is a power differential between the bully and the victim (for additional details see Shakoor et al., 2012[8]). Bullying victimization was reported for 197 of the children.

Lived in foster care. Information from Life History Calendars was used to assess whether children had lived in foster care up to age 12 years. Living in foster care was reported for 24 children.

Low socioeconomic status. Low socioeconomic status at age 5 was assessed based on mother reports, and the variable was coded as a dichotomous indicator of approximately the top quarter of socioeconomic disadvantage, defined as follows: (a) head of household had no educational qualifications; (b) head of household was employed in an unskilled occupation or was not in the labor force; (c) total household gross annual income was less than £10,000; (d) family was receiving at least one government benefit, excluding disability benefit; (e) family housing was government subsidized; and (f) family had no access to a vehicle (for additional details see Kim-Cohen et al., 2004[9]). Low socioeconomic status was indicated for 442 of the children.

© 2019 Rasmussen LJH et al. JAMA Pediatrics.

Peer substance abuse. Peer substance abuse was reported by participants at age 12 and coded as a dichotomous indicator of approximately the top quarter of scores on a scale of five items. The items capture, for each twin separately, the number of peers who drink alcohol, smoke cigarettes, use hash or cannabis, use pharmaceuticals, or sniff glue/gas. Participants responded with none, some, most, or all. Peer substance abuse was indicated for 421 of the participants.

Low parental monitoring (mother). Mothers were asked about their parental monitoring during the last 6 months, and low parental monitoring was coded as a dichotomous indicator of approximately the top quarter of scores on a scale of ten items from the Monitoring and Supervision Questionnaire.[10] The items capture, for each twin separately, whether the child needed permission to leave home or before deciding what to do on the weekend, and whether they had to report on where and who they go out with. Mothers also reported on whether they knew the friends their child hangs out with, where they go in their spare time, how they spend their money, what type of homework or tests and projects they have, and how their child performs in different subjects. Answers were recorded as "no, never" (0), "sometimes" (1), and "yes, always" (2) (for additional details see Wertz et al., 2016[11]). Mother-reported low parental monitoring was indicated for 396 of the participants.

Low parental monitoring (participant). Participants were asked about parental monitoring at age 12 years, and the variable was coded as a dichotomous indicator of approximately the top quarter of scores, using the same items used with mother-rated parental monitoring but worded slightly differently (e.g., "Do your parents know…") (for additional details see Wertz et al., 2016[11]). Participant-reported low parental monitoring was indicated for 475 of the children.

Participant-perceived unsafe neighborhood. At age 12 years, participants reported whether they felt unsafe in their neighborhood by responding to the question: "You feel unsafe in your neighborhood" with true or false. Participant-perceived unsafe neighborhood was reported for 260 participants.

High neighborhood victimization. Neighborhood victimization was assessed in a neighbor survey when participants were age 13–14 years and coded as a dichotomous indicator of approximately the top quarter of scores on a scale of three items tapping into neighbor victimization experiences. Neighbors in the same postal code as the participant were surveyed on whether they had been a victim of 3 different types of crime (home break-in, theft from outdoor home property, violence experienced by respondent or family member in neighborhood). Neighbors were able to respond with "no", "yes, once", and "yes, more than once", for each of the three types of victimization. (Complete details on the survey methodology can be found in Odgers et al., 2009[12]). High neighborhood victimization was indicated for 542 of the participants.

Neighborhood rated unsafe. The participant's neighborhood was rated in a systemic social observation (SSO). Raters used Google Street View images of each participant's neighborhood to respond to two questions on the neighborhood's appearance: whether the raters felt that the neighborhood was "a safe place to live?" and "somewhere they would feel safe walking at night?". Raters provided scores ranging from definitely safe (1) to definitely unsafe (5). (Complete details about the SSO methodology can be found in Odgers et al. 2009[13]). Unsafe neighborhoods were coded as a dichotomous indicator of approximately the top quarter of the average scores of the two items. Unsafe neighborhood was indicated for 410 of the participants.

High-crime neighborhood. Crime in the participants' neighborhoods was assessed using police data. Local area crime was measured by mapping a 1 mile radius around each E-Risk Study family's home and tallying the total number of crimes that occurred in the area each month. Street-level crime data, including information on the type of crime, date of occurrence, and approximate location, were accessed online as part of an open data sharing effort about crime and policing in England and Wales (https://data.police.uk/). An Application Program Interface (API) was used to extract street-level crime data for each of the geospatial coordinates marking the family's home. For a full description see: https://data.police.uk/about/#location-anonymisation. The monthly average of the total number of crimes for the area surrounding each Study family's home was computed for 2011, the first year for which full street-level data was available. High-crime neighborhood was coded as a dichotomous indicator of the top quarter of crime-ridden areas. High-crime neighborhood was indicated for 534 of the participants.

© 2019 Rasmussen LJH et al. JAMA Pediatrics.

eMethods 2. Assessment of severe childhood experiences of stress or violence

We have previously published evidence on the reliability and validity of our measurement of childhood victimization (in the present article termed "severe childhood experiences of stress or violence").[14] Here we summarize the method.

A team of interviewers visited each family at home when the twins reached ages 5, 7, 10, and 12 years. Each home-visit interview was guided by a series of questions in a booklet. Based on these interviews with the mothers, each interviewer coded in the booklet her initial impression of whether or not she thought a child had been maltreated. The interviewers also recorded notes about their experiences in the home, and if an interviewer was worried about a child, she met with the fieldwork coordinator to debrief. Sometimes, the Study had to make a referral to help a child. Codes, notes, and the fieldwork coordinator's narratives from the debriefs have been saved over the years to create a dossier for each child with cumulative information about exposure to domestic violence between the mother and her partner; frequent bullying by peers; physical abuse by an adult; sexual abuse; emotional abuse and neglect; and physical neglect. All the component measures are outlined briefly below.

Physical domestic violence. Mothers reported about perpetration of and victimization by 12 forms of physical violence (e.g., slapping, hitting, kicking, strangling) from the Conflict Tactics Scale,[15] on three assessment occasions during the child's first decade of life (when the children were 5, 7, and 10 years of age). Reports of either perpetration or victimization constituted evidence of physical domestic violence. Families in which no physical violence took place were coded as 0 (55.2%); families in which physical violence took place on one occasion were coded as 1 (28.0%); and families in which physical violence took place on multiple occasions were coded as 2 (16.8%).

Bullying by peers. Experiences of victimization by bullies were assessed using both mothers' and children's reports. During the interview, the following standard definition of bullying was read out: "Someone is being bullied when another child (a) says mean and hurtful things, makes fun, or calls a person mean and hurtful names; (b) completely ignores or excludes someone from their group of friends or leaves them out on purpose; (c) hits, kicks, or shoves a person, or locks them in a room; (d) tells lies or spreads rumors about them; and (e) other hurtful things like these. We call it bullying when these things happen often, and when it is difficult to make it stop. We do not call it bullying when it is done in a friendly or playful way." Mothers were interviewed when children were 7, 10, and 12 years old and asked whether either twin had been bullied by another child, responding never, yes, or frequently. We combined mothers' reports at child age 7 and 10 to derive a measure of victimization during primary school. Mothers' reports when the children were 12 years old indexed victimization during secondary school. During private interviews with the children when they were 12 years old, the children indicated whether they had been bullied by another child during primary or secondary school. When a mother or a child reported victimization, the interviewer asked them to describe what happened. Notes taken by the interviewers were later checked by an independent rater to verify that the events reported could be classified as instances of bullying operationally defined as evidence of (a) repeated harmful actions, (b) between children, and (c) where there is a power differential between the bully and the victim.[16]

Although inter-rater reliability between mothers and children was only modest (kappa = 0.20–0.29), reports of victimization from both informants were similarly associated with children's emotional and behavioral problems, suggesting that each informant provides a unique but meaningful perspective on bullying involvement.[16] We thus combined mother and child reports of victimization to capture all instances of bullying victimization for primary and secondary school separately: reported as not victimized by both mother and child; reported by either mother or child as being occasionally victimized; and reported as being occasionally victimized by both informants or as frequently victimized by either mother or child or both.[17] We then combined these primary and secondary school ratings to create a bullying victimization variable for the entire childhood period (5–12 years). Children who were never bullied in primary or secondary school or occasionally bullied during one of these time periods were coded as 0 (55.5%); children who were occasionally bullied during primary and secondary school, or frequently bullied during one of these time periods were coded as 1 (35.6%); and children who were frequently bullied at both primary and secondary school were coded as 2 (8.9%).

Physical and sexual abuse by an adult. We assessed childhood physical and sexual harm in the E-Risk Study using an approach that resembles the process undertaken by child protection agencies. Essentially this is a two-stage process. In child protection, professionals such as teachers working with children typically raise concerns

© 2019 Rasmussen LJH et al. JAMA Pediatrics.

if they observe signs or symptoms or if they become aware of risk that children are victims of violence. When concerns are raised, child protection officers then review the concerns and evaluate them in the context of information previously gathered on that child or family in order to determine the likelihood that abuse has taken place. In the E-Risk Study, research workers visited the home in pairs, and were extensively trained to detect signs of abuse or neglect. Each time the two research workers visited a home, they interviewed the mother using a structured interview about child harm, tested the children, and observed the family environment using the Home Observation for Measurement of the Environment (HOME).[18] If either research worker had any concerns, they flagged up the case for review. Immediately after each home visit, a review was performed if a family was flagged. In addition, at each wave, any family who had been flagged on a prior wave of the study was automatically reviewed again. The reviews were performed independently by at least 2 clinical psychologists or psychiatrists, and were based on comprehensive dossiers compiled across multiple home visits for each study member during the course of the ongoing longitudinal study. When the twins were aged 5, 7, 10, and 12 their mothers were interviewed about each twin's experience of intentional harm by an adult. At age 5 we used the standardized clinical protocol from the MultiSite Child Development Project.[19,20] At ages 7, 10, and 12 this interview was modified to expand its coverage of contexts for child harm. Interviews were designed to enhance mothers' comfort with reporting valid child maltreatment information, while also meeting researchers' responsibilities for referral under the U.K. Children Act. Specifically, mothers were asked whether either of their twins had been intentionally harmed (physically or sexually) by an adult or had contact with welfare agencies. If caregivers endorsed a question, research workers made extensive notes on what had happened, and indicated whether physical and/or psychological harm had occurred. Under the U.K. Children Act, our responsibility was to secure intervention if maltreatment was current and ongoing. Such intervention on behalf of E-Risk families was carried out with parental cooperation in all but one case. No families left the study following intervention.

Over the years of data collection, the study developed a cumulative profile for each child, comprising the caregiver reports, recorded debriefings with research workers who had coded any indication of maltreatment at any of the successive home visits, recorded narratives of the successive caregiver interviews, and information from clinicians whenever the Study team made a child-protection referral. Each time we visited a home, the research workers flagged concerns, and if there was sufficient evidence to code definite harm then, we did so. If evidence only met the level of probable harm, we kept an "ongoing concern list" and if, at a later wave, there was continued evidence of probable harm, or new evidence, the code was upgraded to definite harm. The profiles were reviewed at the end of the age-12 phase by at least two clinical psychologists or psychiatrists. Inter-rater agreement between the coders was 90% of cases for whom maltreatment was identified (100% for cases of sexual abuse), and discrepantly coded cases were resolved by consensus review. These were coded as: 0 = no physical harm at any age; 1 = probable physical harm at any age; and 2 = definite physical harm at any age. There were 15.0% of children coded as probably being exposed to physical harm and 5.1% as definitely physically harmed by 12 years of age. There were 1.5% of the children coded as being exposed to sexual abuse.

Emotional abuse and neglect. These forms of maltreatment were coded from research workers' narratives of home visit at ages 5, 7, 10, and 12. We coded quite severe examples of parental behavior observed. For example, a mother who had schizophrenia screamed and swore at the children throughout the home visit. As another example, a father who was drunk during the home visit repeatedly spoke abusively to the children in front of the research workers. We found that coders could not empirically separate emotional abuse and emotional neglect in a reliable way and thus such experiences were coded together as emotional abuse/neglect. Inter-rater agreement between the coders exceeded 85% for cases with such emotional abuse/neglect, and discrepant cases were resolved by consensus review. Children with no evidence of emotional abuse/neglect were coded as 0 (88.3%), those where there was some indication of emotionally inappropriate/potentially abusive behavior were coded as 1 (8.7%), and where there was evidence of severe emotional abuse/neglect the children were coded as 2 (3.0%).

Physical neglect. The cumulative observations of the physical state of the home environment documented by the research workers during home visits to the twins at ages 5, 7, 10, and 12 were reviewed by two raters for evidence of physical neglect. This was defined as any sign that the caretaker was not providing a safe, sanitary, or healthy environment for the child. This included the child not having proper clothing or food, as well as grossly unsanitary home environments. (However, this did not include a family living in a crime-ridden neighborhood for economic reasons.) Inter-rater agreement between the coders was 85%, and discrepantly coded cases were resolved by consensus review. Children with no evidence of physical neglect were coded as 0 (90.9%), those for whom there was an indication of minor physical neglect were coded as 1 (7.1%), and where there was evidence of severe physical neglect the children were coded as 2 (2.0%).

© 2019 Rasmussen LJH et al. JAMA Pediatrics.

Childhood poly-victimization. Finkelhor et al. operationalize poly-victimization as the total number of victimization types that a child experiences.[21] The E-Risk poly-victimization variable was derived by summing all victimization experiences that received a code of '2'. Among children in this article, 1,004 (72.2%) children had no severe victimization experiences, 298 (21.4%) had one, 59 (4.2%) had two, and 30 (2.2%) had three or more.

eMethods 3. Assessment of severe adolescent experiences of stress or violence

We have previously published evidence on the reliability and validity of our measurement of adolescent victimization (in this article termed "severe adolescent experiences of stress or violence").[22] Here we summarize the method.

Within each pair of twins in our cohort, co-twins were interviewed separately at age 18 by a different research worker and were assured of the confidentiality of their responses. The participants were advised that confidentiality would only be broken if they told the research worker that they were in immediate danger of being hurt, and in such situations the project leader would be informed and would contact the participant to discuss a plan for safety.

Juvenile Victimization Questionnaire 2nd revision (JVQ-R2) interview. Our adapted version of the JVQ-R2 comprised 5 questions asking about maltreatment, 5 about neglect, 7 about sexual victimization, 6 about family violence, 10 about peer/sibling victimization, 3 about cyber victimization, and 9 about crime victimization. Each JVQ-R2 question was asked for the period "since you were 12". Participants were given the option to say "yes" or "no" as to whether each type of victimization had occurred in the reporting period. Research workers could rate each item "maybe" if the participant seemed unsure or hesitant in their response or they were not convinced that the participant understood the question or was paying attention. Items rated as "maybe" were recoded as "no" or "yes" by the rating team based on the notes provided by the research workers. When insufficient notes were available, these responses were recoded conservatively as a "no". Consistent with the JVQ-R2 manual,[23,24] participants were coded as 1 if they reported any experience within each type of victimization category, or 0 if none of the experiences within the category were endorsed. If an experience was endorsed within a victimization category, follow-up questions were asked concerning how old the participant was when it (first) happened, whether the participant was physically injured in the event, whether the participant was upset or distressed by the event, and how long it went on for (by marking the number of years on a Life History Calendar[25]). In addition, the interviewer wrote detailed notes based on the participant's description of the worst event. If multiple experiences were endorsed within a victimization category, the participant was asked to identify and report about their worst experience.

Victimization dossiers. All information from the JVQ-R2 interview was compiled into victimization dossiers. Using these dossiers, each of the seven victimization categories was rated by an expert in victimology and 3 other members of the E-Risk team who were trained on using the rating criteria. Ratings were made using a 6-point scale: 0 = not exposed, then 1-5 for increasing levels of severity. The anchor points for these ratings were adapted from the coding system used for the Childhood Experience of Care and Abuse interview (CECA[26,27]), which has good inter-rater reliability.[27,28] The CECA is a comprehensive semi-structured interview whose standardized coding system attempts to improve the objectivity of ratings by basing them on the coder's perspective (rather than relying on the participant's judgment) and focusing on concrete descriptions rather than perceptions or emotional responses to the questions, together with considering the context in which the adverse experience occurred.

In our adapted coding scheme, the anchor points of the scale differ for each victimization category, with some focused more on the severity of physical injury that is likely to have been incurred during victimization exposure (crime victimization, family violence, maltreatment), while others are more focused on the frequency of occurrence of victimization (peer/sibling victimization and cyber victimization), the physical intrusiveness of the event (sexual victimization), or the pervasiveness of the effects of victimization (neglect). This reflects the different ways in which severity has previously been defined for different types of victimization.[27,29] (Given that our sample comprises twins, we also coded if any of the victimization events experienced by each twin had been perpetrated by their co-twin, as it is possible that growing up with a genetically related, same-age child could increase or decrease sibling victimization rates.) Each twin's dossier was evaluated separately and we did not use information provided in the co-twin's dossier about their own or shared victimization experiences to rate direct or witnessed violence exposure for the target twin.

© 2019 Rasmussen LJH et al. JAMA Pediatrics.

Reliability. High levels of inter-rater reliability were achieved for the severity ratings for all forms of victimization: crime victimization (intra-class correlation coefficient [ICC]=0.89, *P*<.001), peer/sibling victimization (ICC=0.91, *P*<.001), cyber victimization (ICC=0.90, *P*<.001), sexual victimization (ICC=0.87, *P*<.001), family violence (ICC=0.93, *P*<.001), maltreatment (ICC=0.90, *P*<.001), and neglect (ICC=0.74, *P*<.001).

The ratings for each type of victimization were then grouped into three classes: 0 = no exposure (score of 0), 1 = some exposure (score of 1, 2 or 3), and 2 = severe exposure (score of 4 or 5) due to small numbers for some of the rating points. Combining ratings of 4 and 5 is also consistent with previous studies using the CECA, which have collapsed comparable scale values to indicate presence of "severe" abuse (e.g., Bifulco et al., 1994, 1997, 1998;[27,28,30] Fisher et al., 2011[31]).

Adolescent poly-victimization. Adolescent victimization was derived by summing the number of severe adolescent victimization experiences. In this article, 887 (63.8%) of adolescents had no severe victimization experiences, 275 (19.8%) had one, 131 (9.4%) had two, and 98 (7.1%) had three or more.

eMethods 4. Assessment of cumulative stress and violence experiences

We have previously published on the measurement of cumulative victimization (in this article termed "cumulative stress and violence experiences").[32] Here we summarize the method.

We performed a latent class analysis using longitudinal data about childhood and adolescent victimization. Latent class analysis is a person-centered technique that classifies individuals into groups based on a profile of variables, in this case the degree of each participant's exposure (i.e., none, moderate, or severe) to the six types of childhood and seven types of adolescent victimization. The latent class analysis was performed using only participants who experienced at least one form of victimization. It was conducted in MPlus, version 7.4, accounting for clustering of twins within families. The latent class analysis identified three victimized groups: 1) individuals who were exposed primarily to parental intimate-partner violence in childhood (n=254, 15%), 2) those who were primarily victimized by peers and street crime throughout childhood and adolescence (n=412, 24.8%), and 3) those who experienced multiple types of violence in both childhood and adolescence (n=158, 9.5%). 834 individuals were not exposed to childhood or adolescent victimization.

© 2019 Rasmussen LJH et al. JAMA Pediatrics.

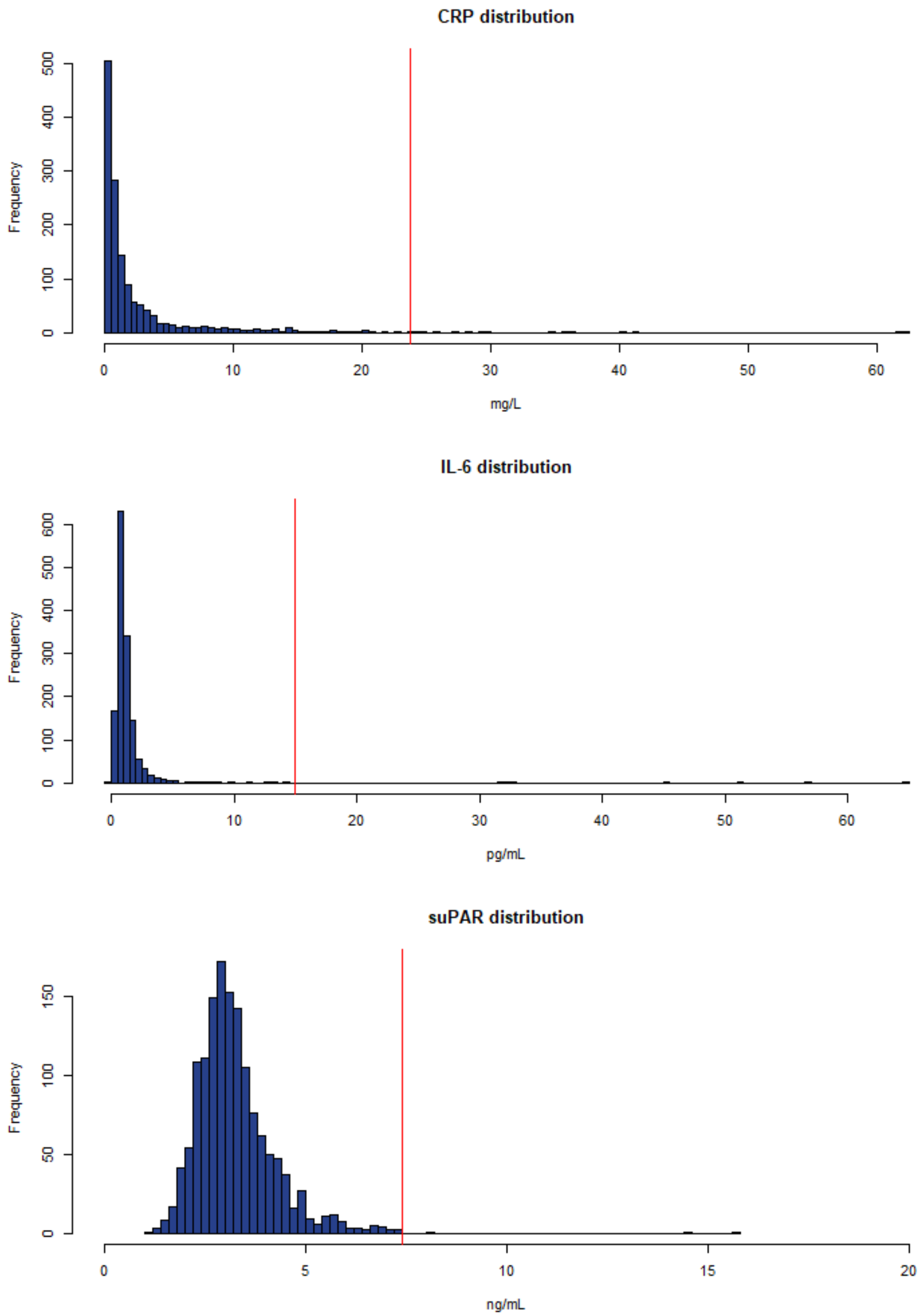

© 2019 Rasmussen LJH et al. JAMA Pediatrics.

eFigure. Distributions of CRP, IL-6, and suPAR in the E-Risk Longitudinal Twin Study. Blood samples were collected at age 18 years. Participants with CRP (n=18), IL-6 (n=7), or suPAR (n=3) levels greater than four standard deviations above the means were excluded; these values are indicated by the red line. CRP and IL-6 were log-transformed to improve normality of their distributions (the figure indicates the distributions of CRP and IL-6 before log-transformation). Abbreviations: CRP, C-reactive protein; IL-6, interleukin-6; suPAR, soluble urokinase plasminogen activator receptor.

© 2019 Rasmussen LJH et al. JAMA Pediatrics.

eTable 1. Correlations (Sex-Adjusted) of Plasma CRP, Plasma IL-6, and Plasma suPAR With Individual Illnesses or Injuries on the Day of Blood Sampling at Age 18 Years in the E-Risk Study (n = 1390)

		CRP[a]		IL-6[a]		suPAR	
**Variable**	***n* (%)**	***r*[b] (95% CI)**	***P***	***r*[b] (95% CI)**	***P***	***r*[b] (95% CI)**	***P***
Fever	9 (0.7)	0.08 (0.03; 0.12)	**<.001**	0.04 (-0.03; 0.10)	.27	0.01 (-0.02; 0.03)	.69
Swollen lymph glands[c]	25 (1.8)	0.09 (0.04; 0.14)	**<.001**	0.04 (0.04; 0.18)	**.0021**	0.03 (0.03; 0.09)	.29
Persistent cough	107 (7.7)	0.20 (0.14; 0.25)	**<.001**	0.17 (0.11; 0.24)	**<.001**	0.04 (-0.01; 0.10)	.10
Cold	155 (11.2)	0.18 (0.13; 0.24)	**<.001**	0.14 (0.07; 0.21)	**<.001**	0.01 (-0.04; 0.06)	.57
Influenza[d]	4 (0.3)	0.09 (0.07; 0.11)	**<.001**	0.10 (0.05; 0.16)	**<.001**	0.01 (-0.01; 0.04)	.39
Asthma[c]	30 (2.2)	0.03 (-0.02; 0.08)	.22	0.04 (-0.02; 0.10)	.23	0.04 (-0.03; 0.10)	.24
Repeated diarrhea	3 (0.2)	-0.002 (-0.03; 0.02)	.88	0.01 (-0.04; 0.05)	.79	0.06 (-0.03; 0.14)	.19
Eye pain/infection	12 (0.9)	0.04 (-0.02; 0.09)	.19	0.01 (-0.03; 0.05)	.66	0.04 (-0.004; 0.09)	.07
Bleeding gums	15 (1.1)	-0.04 (-0.08; 0.003)	.07	0.001 (-0.05; 0.05)	.96	-0.01 (-0.07; 0.05)	.83
Toothache[c]	25 (1.8)	-0.03 (-0.08; 0.02)	.19	0.01 (-0.06; 0.08)	.85	-0.05 (-0.10; -0.0003)	**0.049**
Sore throat	91 (6.6)	0.16 (0.11; 0.21)	**<.001**	0.15 (0.08; 0.21)	**<.001**	0.02 (-0.02; 0.07)	.27
Tonsilitis	7 (0.5)	0.08 (0.04; 0.12)	**<.001**	0.04 (-0.01; 0.08)	.11	0.02 (-0.06; 0.10)	.59
Ear pain/infection	28 (2.0)	0.08 (0.03; 0.13)	**<.001**	0.07 (0.004; 0.13)	**.036**	0.01 (-0.04; 0.06)	.71
Major bruising	10 (0.7)	0.06 (0.01; 0.11)	**.013**	0.01 (-0.03; 0.06)	.55	0.04 (-0.03; 0.10)	.28
Major cuts (incl. tattoos)	5 (0.4)	0.05 (-0.004; 0.10)	.07	0.07 (-0.001; 0.15)	.053	0.06 (0.01; 0.11)	**.015**
Sprains	9 (0.7)	0.02 (-0.04; 0.07)	.54	0.03 (-0.01; 0.07)	.11	0.03 (-0.03; 0.10)	.29

Abbreviations: CI, confidence interval; CRP, C-reactive protein; IL-6, interleukin-6; suPAR, soluble urokinase plasminogen activator receptor.
[a]Log-transformed (natural logarithm)
[b]Standardized estimated regression coefficients; all correlations are adjusted for sex, and *P* values are adjusted for clustering within families.
[c]n=1,389
[c]n=1,388

© 2019 Rasmussen LJH et al. JAMA Pediatrics.

eTable 2. Results of a Latent Class Analysis Using Data About Inflammation Measured With CRP, IL-6, and suPAR (n = 1390)[a]

No. groups	Loglikelihood	AIC	BIC	Entropy	LMR-adjusted LRT	*P* Value
2	-5362.641	10745.282	10797.653	0.860	420.215	0.0002
3	-5269.287	10566.574	10639.893	0.774	180.473	0.0006
4	-5234.261	10504.523	10598.790	0.750	67.713	0.0197
5	-5217.430	10478.861	10594.076	0.727	32.538	0.6556
6	-5198.254	10448.508	10584.671	0.730	37.068	0.0602

Abbreviations: AIC, Akaike information criterion; BIC, Bayesian information criterion; CRP, C-reactive protein; IL-6, interleukin-6; LMR-adjusted LRT Test, Lo-Mendell-Rubin adjusted likelihood ratio test; suPAR, soluble urokinase plasminogen activator receptor.

[a]We examined fit statistics for 2 to 6 groups. The best solution appeared to be either a 3- or 4-class solution, with the entropy test favoring the 3-class solution and the chi-square difference test favoring the 4-class solution. The difference between the solutions is that the 4-class solution divided the final 3-class solution group into participants with ‘high’ and ‘very high’ values on all three inflammation biomarkers. One participant with extreme values was excluded from the Latent Class Analysis.

© 2019 Rasmussen LJH et al. JAMA Pediatrics.

eTable 3. Levels of CRP, IL-6, and suPAR in the 3 Inflammation Groups Identified by Latent Class Analysis (n = 1390)

		CRP			IL-6			suPAR	
**Latent class inflammation groups**	**n**	**Median (IQR)**	**Z-score mean (95% CI)**		**Median (IQR)**	**Z-score mean (95% CI)**		**Median (IQR)**	**Z-score mean (95% CI)**
1: Low inflammation	1,057	0.56 (0.27; 1.19)	-0.36 (-0.40; -0.31)		0.79 (0.59; 1.07)	-0.31 (-0.35; -0.26)		2.91 (2.50; 3.39)	-0.27 (-0.31; -0.23)
2: Elevated CRP and IL-6	249	5.55 (3.01; 10.68)	1.25 (1.17; 1.34)		1.82 (1.35; 2.58)	1.14 (1.03; 1.26)		3.40 (3.01; 4.00)	0.26 (0.17; 0.36)
3: Elevated CRP, IL-6, and suPAR	84	2.84 (1.13; 7.56)	0.76 (0.56; 0.96)		1.30 (0.94; 1.93)	0.60 (0.40; 0.81)		5.57 (5.04; 6.05)	2.61 (2.44; 2.77)

Abbreviations: CI, confidence interval; CRP, C-reactive protein; IL-6, interleukin-6; IQR, interquartile range; suPAR, soluble urokinase plasminogen activator receptor.

© 2019 Rasmussen LJH et al. JAMA Pediatrics.

eTable 4. Correlations Between Different Types of Adverse Experiences and CRP, IL-6, and suPAR at Age 18 Years in the E-Risk Study

		CRP[a]			IL-6[a]			suPAR	
**Variable**	**n (%)**	***r*[b] (95% CI)**	***P***		***r*[b] (95% CI)**	***P***		***r*[b] (95% CI)**	***P***
**Age 12 years:**									
Emotional abuse/neglect	45 (3.2)	0.03 (-0.03; 0.08)	.37		0.01 (-0.03; 0.05)	.59		0.02 (-0.02; 0.07)	.30
Foster/non-parental care	18 (1.3)	-0.02 (-0.08; 0.04)	.54		0.01 (-0.03; 0.05)	.54		0.03 (-0.02; 0.07)	.28
Physical abuse	75 (5.4)	0.01 (-0.05; 0.07)	.70		0.05 (-0.003; 0.10)	.06		0.08 (0.01; 0.14)	**.016**
Physical neglect	24 (1.7)	0.03 (-0.03; 0.08)	.36		0.06 (0.001; 0.13)	**.045**		0.05 (-0.02; 0.13)	.17
Sexual abuse	11 (0.8)	0.001 (-0.06; 0.06)	.98		0.04 (-0.03; 0.12)	.25		0.03 (-0.01; 0.07)	.13
**Age 18 years:**									
Crime victimization	265 (19.1)	0.02 (-0.04; 0.07)	.55		0.06 (-0.003; 0.11)	.06		0.11 (0.05; 0.16)	**<.001**
Maltreatment	43 (3.1)	-0.002 (-0.06; 0.06)	.93		0.007 (-0.05; 0.06)	.80		0.04 (-0.01; 0.10)	.13
Peer victimization	226 (16.2)	0.05 (-0.003; 0.11)	.06		0.01 (-0.04; 0.07)	.58		0.05 (-0.01; 0.11)	.08
Sexual victimization (n=1,390)	39 (2.8)	0.02 (-0.02; 0.06)	.37		0.04 (-0.01; 0.10)	.14		0.07 (0.01; 0.13)	**.015**
Family victimization	182 (13.1)	0.04 (-0.02; 0.09)	.16		-0.008 (-0.07; 0.05)	.77		0.07 (0.02; 0.12)	**.0098**
Cyber victimization	93 (6.7)	0.02 (-0.03; 0.07)	.46		0.04 (-0.01; 0.09)	.14		0.01 (-0.05; 0.07)	.73
Neglect	29 (2.1)	0.02 (-0.03; 0.06)	.49		-0.02 (-0.06; 0.03)	.40		0.02 (-0.03; 0.06)	.47

Abbreviations: CI, confidence interval; CRP, high-sensitivity C-reactive protein; IL-6, interleukin-6; suPAR, soluble urokinase plasminogen activator receptor.
[a]Log-transformed (natural logarithm).
[b]Standardized estimated regression coefficients; all correlations are adjusted for sex, with confidence intervals and P values adjusted for clustering within families.

© 2019 Rasmussen LJH et al. JAMA Pediatrics.

eTable 5. Associations of Childhood Adversities With Plasma suPAR at Age 18 Years in the E-Risk Study After Adjustment for Sex and Indicated Correlates

	Adjusted for sex and:									
	Cleanliness of home (n=1,342)		Childhood SES (n=1,391)		CRP[a] (n=1,391)		IL-6[a] (n=1,391)		CRP[a] and IL-6[a] (n=1,391)	
**Victimization measures**	**B (95% CI)[b]**	**β (95% CI)[c]**	**B (95% CI)[b]**	**β (95% CI)[c]**	**B (95% CI)[b]**	**β (95% CI)[c]**	**B (95% CI)[b]**	**β (95% CI)[c]**	**B (95% CI)[b]**	**β (95% CI)[c]**
Adverse childhood experiences	0.05 (0.02 to 0.07)	0.13 (0.06 to 0.20)	0.04 (0.01 to 0.06)	0.10 (0.03 to 0.18)	0.05 (0.03 to 0.07)	0.15 (0.09 to 0.21)	0.05 (0.03 to 0.07)	0.14 (0.08 to 0.20)	0.05 (0.03 to 0.07)	0.14 (0.08 to 0.19)
Severe childhood experience of stress or violence	0.13 (0.05 to 0.22)	0.10 (0.03 to 0.16)	0.13 (0.05 to 0.22)	0.10 (0.03 to 0.16)	0.16 (0.08 to 0.24)	0.12 (0.06 to 0.17)	0.15 (0.06 to 0.23)	0.10 (0.05 to 0.16)	0.14 (0.06 to 0.22)	0.10 (0.05 to 0.16)
Severe adolescent experience of stress or violence	0.09 (0.03 to 0.16)	0.09 (0.03 to 0.16)	0.09 (0.03 to 0.15)	0.09 (0.03 to 0.15)	0.10 (0.04 to 0.16)	0.10 (0.04 to 0.16)	0.10 (0.04 to 0.16)	0.10 (0.04 to 0.16)	0.10 (0.04 to 0.16)	0.10 (0.04 to 0.15)
Groups of cumulative stress and violence experiences (birth to age 18) identified in latent class analysis:										
Exposure to parental intimate-Partner violence in childhood[d]	0.31 (0.15 to 0.48)	0.34 (0.16 to 0.51)	0.28 (0.12 to 0.44)	0.31 (0.13 to 0.48)	0.33 (0.18 to 0.48)	0.36 (0.19 to 0.52)	0.33 (0.18 to 0.49)	0.36 (0.19 to 0.52)	0.32 (0.17 to 0.47)	0.35 (0.19 to 0.51)
Exposure to peer and street-crime victimization throughout childhood and adolescence[d]	0.13 (0.01 to 0.25)	0.14 (0.01 to 0.27)	0.12 (-0.003 to 0.24)	0.13 (-0.003 to 0.26)	0.12 (-0.003 to 0.24)	0.13 (-0.003 to 0.25)	0.13 (0.01 to 0.26)	0.14 (0.01 to 0.28)	0.12 (-0.001 to 0.24)	0.13 (-0.001 to 0.26)
Exposure to multiple types of violence in both childhood and adolescence[d]	0.39 (0.18 to 0.59)	0.42 (0.20 to 0.64)	0.37 (0.16 to 0.57)	0.40 (0.17 to 0.62)	0.43 (0.24 to 0.62)	0.47 (0.26 to 0.67)	0.43 (0.25 to 0.62)	0.47 (0.27 to 0.67)	0.42 (0.24 to 0.61)	0.45 (0.25 to 0.65)

Abbreviations: CI, confidence interval; CRP, C-reactive protein; IL-6, interleukin-6; SES, socioeconomic status; suPAR, soluble urokinase plasminogen activator receptor.

[a]Log-transformed (natural logarithm).

[b]Unstandardized B coefficient for Ordinary Least Squares regression model, where a 1-unit change in the predictor (e.g., adverse childhood experiences) is associated with a corresponding change in B, holding all other variables constant.

[c]Standardized regression coefficients.

© 2019 Rasmussen LJH et al. JAMA Pediatrics.

[d]Estimates represent standardized mean differences from the “No victimization” group.

eTable 6. Associations Between Adverse Experiences and Inflammation Groups at Age 18 Years[a]

**Adverse childhood experiences (ACEs)**						
	**OR (95% CI)**	***P***			**OR (95% CI)**	***P***
Low CRP, low suPAR	1			Low IL-6, low suPAR	1	
High CRP, low suPAR	1.03 (0.87–1.23)	.71		High IL-6, low suPAR	1.12 (0.96–1.31)	0.14
Low CRP, high suPAR	1.22 (1.03–1.43)	**.019**		Low IL-6, high suPAR	1.26 (1.07–1.49)	**0.0069**
High CRP, high suPAR	1.41 (1.16–1.71)	**<.001**		High IL-6, high suPAR	1.39 (1.13–1.71)	**0.0020**
**Severe childhood experience of stress or violence**						
	**OR (95% CI)**	***P***			**OR (95% CI)**	***P***
Low CRP, low suPAR	1			Low IL-6, low suPAR	1	
High CRP, low suPAR	1.09 (0.92–1.30)	.32		High IL-6, low suPAR	1.18 (1.01–1.38)	**.036**
Low CRP, high suPAR	1.15 (0.98–1.36)	.088		Low IL-6, high suPAR	1.18 (1.01–1.38)	**.040**
High CRP, high suPAR	1.33 (1.12–1.57)	**.0010**		High IL-6, high suPAR	1.34 (1.11–1.63)	**.0027**
**Severe adolescent experience of stress or violence**						
	**OR (95% CI)**	***P***			**OR (95% CI)**	***P***
Low CRP, low suPAR	1			Low IL-6, low suPAR	1	
High CRP, low suPAR	1.15 (0.99–1.35)	.071		High IL-6, low suPAR	1.08 (0.92–1.28)	.36
Low CRP, high suPAR	1.33 (1.14–1.55)	**<.001**		Low IL-6, high suPAR	1.27 (1.09–1.48)	**.0025**
High CRP, high suPAR	1.28 (1.05–1.56)	**.015**		High IL-6, high suPAR	1.35 (1.12–1.64)	**.0022**
**Cumulative stress or violence experiences: Multiple types of violence**						
	**OR (95% CI)**	***P***			**OR (95% CI)**	***P***
Low CRP, low suPAR	1			Low IL-6, low suPAR	1	
High CRP, low suPAR	1.37 (0.75–2.48)	.31		High IL-6, low suPAR	1.13 (0.62–2.04)	.70
Low CRP, high suPAR	2.45 (1.39–4.30)	**.0020**		Low IL-6, high suPAR	2.04 (1.13–3.71)	**.019**
High CRP, high suPAR	2.51 (1.27–4.97)	**.0080**		High IL-6, high suPAR	3.04 (1.61–5.73)	**<.001**

© 2019 Rasmussen LJH et al. JAMA Pediatrics.

Abbreviations: CI, confidence interval; CRP, C-reactive protein; IL-6, interleukin-6; OR, odds ratio; suPAR, soluble urokinase plasminogen activator receptor.
[a]Standardized odds ratios with confidence intervals and P values adjusted for clustering within families.

© 2019 Rasmussen LJH et al. JAMA Pediatrics.

eTable 7. Associations Between Adverse Experiences and Latent Class Inflammation Groups at Age 18 Years[a]

Latent class inflammation groups	OR (95% CI)	*P*
**Adverse childhood experiences (ACEs)**		
**Latent class inflammation groups**	**OR (95% CI)**	***P***
1: Low inflammation	1	
2: Elevated CRP and IL-6	1.12 (0.97–1.28)	.12
3: Elevated CRP, IL-6, and suPAR	1.38 (1.11–1.72)	**.0036**
**Severe childhood experience of stress or violence**		
**Latent class inflammation groups**	**OR (95% CI)**	***P***
1: Low inflammation	1	
2: Elevated CRP and IL-6	1.13 (0.97–1.30)	.11
3: Elevated CRP, IL-6, and suPAR	1.30 (1.06–1.60)	**.011**
**Severe adolescent experience of stress or violence**		
**Latent class inflammation groups**	**OR (95% CI)**	***P***
1: Low inflammation	1	
2: Elevated CRP and IL-6	1.12 (0.97–1.29)	.14
3: Elevated CRP, IL-6, and suPAR	1.34 (1.08–1.67)	**.0089**
**Cumulative stress or violence experiences: Multiple types of violence**		
**Latent class inflammation groups**	**OR (95% CI)**	***P***
1: Low inflammation	1	
2: Elevated CRP and IL-6	1.10 (0.63–1.93)	.74
3: Elevated CRP, IL-6, and suPAR	4.07 (2.10–7.90)	**<.001**

Abbreviations: CI, confidence interval; CRP, C-reactive protein; IL-6, interleukin-6; OR, odds ratio; suPAR, soluble urokinase plasminogen activator receptor.
[a]Standardized odds ratios with confidence intervals and P values adjusted for clustering within families.

© 2019 Rasmussen LJH et al. JAMA Pediatrics.

eReferences

1. Beckley AL, Caspi A, Arseneault L, et al. The Developmental Nature of the Victim-Offender Overlap. *J Dev Life Course Criminol*. 2018;4(1):24-49.

2. Felitti VJ, Anda RF, Nordenberg D, et al. Relationship of Childhood Abuse and Household Dysfunction to Many of the Leading Causes of Death in Adults. The Adverse Childhood Experiences (ACE) Study. *Am J Prev Med*. 1998;14(4):245-258.

3. Achenbach TM. *Young Adult Self Report*. Burlington, VT: University of Vermont, Department of Psychiatry; 1997.

4. Jaffee SR, Moffitt TE, Caspi A, Taylor A. Life with (or without) father: the benefits of living with two biological parents depend on the father's antisocial behavior. *Child Dev*. 2003;74(1):109-126.

5. Caspi A, Taylor A, Smart M, Jackson J, Tagami S, Moffitt TE. Can women provide reliable information about their children's fathers? Cross-informant agreement about men's lifetime antisocial behaviour. *J Child Psychol Psychiatry*. 2001;42(7):915-920.

6. Milne BJ, Moffitt TE, Crump R, et al. How should we construct psychiatric family history scores? A comparison of alternative approaches from the Dunedin Family Health History Study. *Psychol Med*. 2008;38(12):1793-1802.

7. Belsky DW, Caspi A, Arseneault L, et al. Etiological features of borderline personality related characteristics in a birth cohort of 12-year-old children. *Dev Psychopathol*. 2012;24(1):251-265.

8. Shakoor S, Jaffee SR, Bowes L, et al. A prospective longitudinal study of children's theory of mind and adolescent involvement in bullying. *J Child Psychol Psychiatry*. 2012;53(3):254-261.

9. Kim-Cohen J, Moffitt TE, Caspi A, Taylor A. Genetic and environmental processes in young children's resilience and vulnerability to socioeconomic deprivation. *Child Dev*. 2004;75(3):651-668.

10. Stattin H, Kerr M. Parental monitoring: A reinterpretation. *Child Dev*. 2000;71(4):1072-1085.

11. Wertz J, Nottingham K, Agnew-Blais J, et al. Parental monitoring and knowledge: Testing bidirectional associations with youths' antisocial behavior. *Dev Psychopathol*. 2016;28(3):623-638.

12. Odgers CL, Moffitt TE, Tach LM, et al. The Protective Effects of Neighborhood Collective Efficacy on British Children Growing Up in Deprivation: A Developmental Analysis. *Dev Psychol*. 2009;45(4):942-957.

13. Odgers CL, Bates C, Caspi A, Sampson R, Moffitt TE. *Systematic Social Observation Inventory - Tally of Observations in Urban Regions (SSO i-Tour)*. Adaptlab publications: Irvine, CA; 2009.

14. Danese A, Moffitt TE, Arseneault L, et al. The origins of cognitive deficits in victimized children: Implications for neuroscientists and clinicians. *Am J Psychiatry*. 2017;174(4):349-361.

15. Straus M, Gelles R. *Physical Violence in American Families: Risk Factors and Adaptations to Violence in 8,145 Families*. New Brunswick, N.J., U.S.A.: Transaction Publishers; 1990.

16. Shakoor S, Jaffee SR, Andreou P, et al. Mothers and children as informants of bullying victimization: Results from an epidemiological cohort of children. *J Abnorm Child Psychol*. 2011;39(3):379-387.

17. Bowes L, Maughan B, Ball H, et al. Chronic bullying victimization across school transitions: The role of genetic and environmental influences. *Dev Psychopathol*. 2013;25(2):333-346.

18. Bradley RH, Caldwell BM. Home observation for measurement of the environment: a validation study of screening efficiency. *Am J Ment Defic*. 1977;81(5):417-420.

19. Dodge KA, Bates JE, Pettit GS. Mechanisms in the cycle of violence. *Science*. 1990;250(4988):1678-1683.

© 2019 Rasmussen LJH et al. JAMA Pediatrics.

20. Lansford JE, Dodge KA, Pettit GS, Bates JE, Crozier J, Kaplow J. A 12-year prospective study of the long-term effects of early child physical maltreatment on psychological, behavioral, and academic problems in adolescence. *Arch Pediatr Adolesc Med*. 2002;156(8):824-830.

21. Finkelhor D, Ormrod RK, Turner HA. Poly-victimization: A neglected component in child victimization. *Child Abuse Negl*. 2007;31(1):7-26.

22. Fisher HL, Caspi A, Moffitt TE, et al. Measuring adolescents' exposure to victimization: The Environmental Risk (E-Risk) Longitudinal Twin Study. *Dev Psychopathol*. 2015;27(4 Pt 2):1399-1416.

23. Finkelhor D, Hamby S, Turner H, Ormrod R. *The Juvenile Victimization Questionnaire: 2nd Revision (JVQ-R2)*. Durham, NH: Crimes Against Children Research Center; 2011.

24. Hamby S, Finkelhor D, Ormrod R, Turner H. *The Comprehensive JV Administration and Scoring Manual*. Durham, NH: Crimes Against Children Research Center; 2004.

25. Caspi A, Moffitt TE, Thornton A, et al. The Life History Calendar: A Research and clinical assessment method for collecting retrospective event-history data. *Int J Methods Psychiatr Res*. 1996;6(2):101-114.

26. Bifulco A, Brown G, Neubauer A, Moran P, Harris T. *Childhood Experience of Care and Abuse (CECA) Training Manual*. London: Royal Holloway College, University of London; 1994.

27. Bifulco A, Brown GW, Harris TO. Childhood Experience of Care and Abuse (CECA): a retrospective interview measure. *J Child Psychol Psychiatry*. 1994;35(8):1419-1435.

28. Bifulco A, Brown GW, Lillie A, Jarvis J. Memories of childhood neglect and abuse: Corroboration in a series of sisters. *J Child Psychol Psychiatry*. 1997;38(3):365-374.

29. Barnett D, Manly JT, Cicchetti D. Defining child maltreatment: The interface between policy and research. In: Cichetti D, Toth L, eds. *Child Abuse, Child Development, and Social Policy*. Norwood, NJ: Ablex; 1993:7-74.

30. Bifulco A, Brown GW, Moran P, Ball C, Campbell C. Predicting depression in women: the role of past and present vulnerability. *Psychol Med*. 1998;28(1):39-50.

31. Fisher HL, Bunn A, Jacobs C, Moran P, Bifulco A. Concordance between mother and offspring retrospective reports of childhood adversity. *Child Abuse Negl*. 2011;35(2):117-122.

32. Marzi SJ, Sugden K, Arseneault L, et al. Analysis of DNA methylation in young people: Limited evidence for an association between victimization stress and epigenetic variation in blood. *Am J Psychiatry*. 2018;175(6):517-529.

© 2019 Rasmussen LJH et al. JAMA Pediatrics.
